# An Internal Consistency Reliability Study of the Catalyst Datafinch Applied Behavior Analysis Data Collection Application With Autistic Individuals

**DOI:** 10.7759/cureus.58379

**Published:** 2024-04-16

**Authors:** Tami Peterson, Jessica Dodson, Robert Sherwin, Frederick Strale

**Affiliations:** 1 Hyperbaric Oxygen Therapy, The Oxford Center, Brighton, USA; 2 Applied Behavior Analysis, The Oxford Center, Brighton, USA; 3 Hyperbaric Oxygen Therapy, Wayne State University School of Medicine, Detroit, USA; 4 Biostatistics, The Oxford Center, Brighton, USA

**Keywords:** interclass correlations, split-half reliability, inter-item correlations, cronbach’s alpha, internal consistency reliability, psychometrics tests

## Abstract

Introduction

Many psychometric studies have scrutinized the dependability of different instruments for evaluating and treating autism using applied behavior analysis (ABA). However, there has been no exploration into the psychometric attributes of the Catalyst Datafinch Applied Behavior Analysis Data Collection Application, namely, internal consistency reliability measures.

Materials and methods

Four datasets were extracted (n=100, 98, 103, and 62) from published studies at The Oxford Center, Brighton, MI, ranging from March 19, 2023, through January 8, 2024, using Catalyst Datafinch as the data collection tool. All data were gathered by Board Certified Behavior Analysts (BCBAs) and behavioral technicians and designed to replicate how practitioners collect traditional paper and pencil data. SPSS Statistics (v. 29.0) computed internal consistency reliability measures, including Cronbach’s alpha, inter-item, split-half, and interclass correlation coefficients.

Results

Dataset #1: Cronbach’s alpha was 0.916 with seven items, indicating excellent reliability. Cronbach's split-half reliability for Part 1 was 0.777, indicating good reliability, and for Part 2 was 0.972, indicating excellent reliability. Guttman split-half coefficient was 0.817, indicating good reliability. Inter-item correlation coefficients ranged from 0.474 to 0.970. The average measures interclass correlation (ICC) was 0.916, indicating excellent reliability. Single measures (ICC) reliability was 0.609, indicating acceptable reliability. Dataset #2: Cronbach’s alpha was 0.954 with three items, indicating excellent reliability. Cronbach's split-half reliability for Part 1 was 0.912, indicating excellent reliability, and for Part 2 was 0.975, indicating excellent reliability. Guttman split-half coefficient was 0.917, indicating excellent reliability. Inter-item correlation coefficients ranged from 0.827 to 0.977. Average measures (ICC) was 0.954, indicating excellent reliability. Single measures (ICC) reliability was 0.875, indicating good reliability. Dataset #3: Cronbach’s alpha was 0.974 with three items, indicating excellent reliability. Cronbach's split-half reliability for Part 1 was 0.978, indicating excellent reliability. Split-half reliability for Part 2 was 0.970, indicating excellent reliability. Guttman split-half coefficient was 0.935, indicating excellent reliability. Inter-item correlation coefficients ranged from 0.931 to 0.972. The average measures (ICC) was 0.974, indicating excellent reliability. Single measures (ICC) reliability was 0.926, indicating excellent reliability. Dataset #4: Cronbach’s alpha was 0.980 with 12 items, indicating excellent reliability. Cronbach's split-half reliability for Part 1 was 0.973, indicating excellent reliability. Split-half reliability for Part 2 was 0.996, indicating excellent reliability. Guttman split-half coefficient was 0.838, indicating good reliability. Inter-item correlation coefficients ranged from 0.692 to 0.999. The average measures (ICC) was 0.980, indicating excellent reliability. Single measures (ICC) reliability was 0.804, indicating good reliability.

Conclusions

These results suggest that Catalyst Datafinch demonstrates high internal consistency reliability when used with individuals with autism. This indicates that the application is reliable for collecting and analyzing behavioral data in this population. The ratings ranged from good to excellent, indicating a high consistency in the measurements.

## Introduction

A variety of evaluation tools exist to identify and measure the primary symptoms and behavioral results in individuals with autism spectrum disorder (ASD). There are frequently used instruments in randomized trials or ongoing registries to gauge health outcomes for individuals with ASD. While many of these tools are employed in clinical environments to characterize autism symptoms and disabilities or to determine the disorder’s severity for diagnostic reasons, they have been and continue to be utilized as outcome indicators in research contexts, particularly in clinical trials [1]. In their systematic review and meta-analysis, Yu et al. [2] pointed out general measures of symptomatic outcomes of ASD, including outcomes for socialization, communication, expressive language, receptive language, adaptive behavior, daily living skills, and intelligence quotient. 

Typical paper and pencil measures for general symptom outcomes of ASD include the Mullen Scales of Early Learning (MSEL) [3], Autism Diagnostic Observation Schedule (ADOS) [4], Assessment of Basic Language and Learning Skills (ABLLS) [5], Aberrant Behavior Checklist (ABC) [6], Autism Diagnostic Interview-Revised (ADI-R) [7], Vineland Adaptive Behavior Scales (VABS) [8], Autism Treatment Evaluation Checklist (ATEC) [9], and Childhood Autism Rating Scale (CARS) [10]. ADI-R [7] and VABS [8] were selected for socialization outcomes. VABS and Psychoeducational Profile (C-PEP) [11] were chosen for communication outcomes. MSEL [3], ADOS [4], and Reynell Developmental Language Scales (RDLS) [12] were selected for expressive language outcomes. RDLS [12] and MSEL [3] were chosen for receptive language outcomes. VABS [8] and C-PEP [11] were selected for adaptive behavior outcomes. VABS [8] was also used to assess daily living skills. Lastly, Differential Ability Scales (DAS) [13] and Stanford-Binet Intelligence Scale (SBIS) [14] were selected as measures for IQ outcomes. However, Catalyst Datafinch does not explicitly track and measure intelligence variables.

In the 1990s, many tracking methods were created, such as manual tools using paper and pencil. They were completed by parents, caregivers, or behavioral therapists to monitor progress or lack thereof. However, with the advent and integration of electronic tools in the treatment outcomes for children with autism, these instruments have become indispensable.

Early Intensive Behavioral Interventions (EIBI), a proven and effective treatment method for children with autism, heavily depends on data for progress evaluation [15]. These electronic tools can facilitate this process by offering a simplified and efficient data collection and analysis method, ultimately contributing to improved treatment results. It is important to remember that, while these tools can significantly assist in treatment, they are merely one component of a comprehensive approach to autism treatment. They should be used with other strategies and interventions for a well-rounded treatment plan.

Catalyst Datafinch 

Catalyst is a data collection instrument developed by DataFinch Technologies and is a market-ready tool for electronic data collection. It was designed to aid applied behavior analysts in capturing and analyzing extensive datasets in the context of autism spectrum and related developmental disorders. Users of Catalyst, such as Board-Certified Behavior Analysts (BCBAs) and behavioral technicians, set up a unique profile for each autistic patient and devise programs and data collection methods for managing problem behavior and skill acquisition programs [16].

Data are collected using a real-time data-stamping method, allowing for a detailed examination of the data down to the exact second it was collected rather than just a summary metric. For problem behavior, users define the problem operationally, choose from a variety of continuous (e.g., frequency, duration) and discontinuous measurement systems, and set the interval length for discontinuous systems, namely 10 seconds, 30 seconds, and two minutes from a drop-down menu in the portal [16].

All problem behavior topography that uses the same discontinuous measurement system shares the same interval setting, which the patient, not the topography, determines. Typically, technicians, such as registered behavior technicians, use a portable electronic device, usually an iPad, to record data during ongoing therapy sessions [16]. When a discontinuous measurement system is in use, an auditory or vibratory stimulus (user-selected setting) indicates the end of the interval. The technician then records whether each problem behavior is happening or has occurred since the last signal or for the entire interval [16].

Scientific discourse calls for the significant need for psychometric studies that delineate the reliability and validity of instruments designed to measure behaviors and skill acquisition in individuals with autism [17]. Reliability is a crucial aspect of any psychological measurement instrument [18]. They ensure that the tool consistently measures what it intends to measure. In the context of autism, this is particularly important due to the wide range of behaviors and skills that can be present in this population [17,18]. Psychometric studies can help improve assessment. They can enhance the accuracy of assessments, leading to more effective intervention strategies. They can help tailor interventions to the individual needs of each autistic person. They can provide a reliable means of tracking progress over time, which is crucial for evaluating the effectiveness of interventions. Without these studies, there is a risk that the tools used may not accurately capture the complexities of behaviors and skill acquisition in individuals with autism. This could lead to misinterpretation of data, ineffective interventions, and missed opportunities for skill development. Therefore, ongoing psychometric research is essential [17-19].

While numerous psychometric studies have examined the reliability of various tools used in assessing and treating autism with ABA [20-22], no research studies have looked at the psychometric properties of the Catalyst Datafinch application. This study addresses internal consistency reliability measures, assessing Cronbach’s alpha, inter-item, split-half, and interclass correlation coefficients.

## Materials and methods

Four datasets were extracted (n=100, n=98, n=103, n=62) from previously published studies at The Oxford Center, Brighton and Troy, MI [23-26], ranging from March 19, 2023, through January 8, 2024, using Catalyst Datafinch as the data collection tool. All data were gathered by Board Certified Behavior Analysts (BCBAs) and behavioral technicians and designed to replicate how practitioners collect traditional paper and pencil data. Internal consistency reliability measures were computed and reported, including Cronbach’s alpha, inter-item, split-half, and interclass correlation coefficients.

Dataset #1 

General target mastery data were collected daily by a team of multiple (three to five) behavioral technicians per child for 100 individuals with autism via retrospective chart reviews [23]. Behavior analysts administered a mixed model of discrete trial training, mass trials, and naturalistic environment treatment for three months between March 19, 2023, and June 11, 2023. Data were obtained at two-week intervals for seven time points (baseline, two weeks, four weeks, six weeks, eight weeks, 10 weeks, and 12 weeks). General target mastery data were collected for 89 children and four adults, with seven missing values [23]. Behavior technicians assigned to specific autistic individuals used real-time data-stamping procedures to enter data the second the behavior was observed. The behavior technician created an operational definition for the problem behavior and selected continuous (frequency, duration) measurement systems using a portable electronic device (an iPad; Apple Inc., Cupertino, CA). Researchers then accessed those data online for analysis and reporting [23].

*Sample Demographics* 

For the sample of 100 autistic individuals, the age was M=8.8817 and SD=8.05, the median was 7, the minimum was 1, and the maximum was 73. There were seven missing values. There were 74 males (74.0%) and 25 females (25.0%), with one missing value. There were 75 Whites (75.0%), four Asians (4.0%), three Hispanics (4.0%), 12 Middle Eastern (12.0%), and six African Americans (6.0%), with no missing values [24]. In terms of age categories, 18 (18.0%) were in the one to four-year category, 39 (39.0%) were in the five to eight-year category, 20 (20.0%) were in the 9-12-year category, 12 (12.0%) were in the 13-16-year category, and four (4.0%) were in the 17-73-year category. Four subjects were over 17 years old, i.e., 18 years, 20 years, 25 years, and 73 years. There were seven missing values [23].

Dataset #2 

Target mastery data were collected for 98 autistic individuals, including four adults over 18 years of age, via a retrospective chart review who were administered ABA treatment for one month between June 7, 2023, and July 7, 2023. Data were obtained at two-week intervals for three time points (baseline, two weeks, and four weeks) [24]. Behavior technicians assigned to specific individuals with autism utilized procedures that allowed for data entry in real time the moment a behavior was observed. These technicians established a clear, operational definition for the problematic behavior. They chose to use continuous measurement systems (tracking frequency and duration) with the help of a portable electronic device, specifically an iPad. These data were then accessible online for researchers to analyze and report on [24].

*Sample Demographics* 

For the sample of 98 autistic children, the age was M=9.0 and SD =8.15, the median was 7.5, the minimum was 1, and the maximum was 73. There were 70 males (71.4%) and 25 females (25.5%), with three (3.1%) missing values. There were 68 Whites (69.4%), 12 Asians (12.2%), five American Indian/Alaska Native (5.1%), four Hispanics (4.1%), and seven unspecified (7.1%), with two (2.0%) missing values [25]. Regarding age categories, 17 (17.3%) were in the 1-4-year category, 37 (37.8%) were in the 5-8-year category, 20 (20.4%) were in the 9-12-year category, 12 (12.2%) were in the 13-16-year category, and four (4.1%) were in the 17-73-year category, with eight (8.2%) missing values. Please note that four subjects were over 17 years old, e.g., 18 years old, 20 years old, 25 years old, and 73 years old [24].

Dataset #3 

Participant cohort target mastery data were gathered using a retrospective chart review from 103 autistic individuals who received ABA treatment. A repeated measures analysis covered three time points (baseline, two weeks, and four weeks) between June 7, 2023, and August 8, 2023, measuring cumulative target behaviors [25]. Behavior technicians tasked with monitoring specific individuals with autism employed real-time data entry methods, recording observations as they occurred. They formulated a detailed, operational definition for the behavior deemed problematic. They opted for continuous measurement systems (monitoring both frequency and duration) using a portable electronic device, an iPad. The collected data were subsequently made available online, enabling researchers to conduct their analysis and compile their reports [25].

Sample Demographics

For the sample of 103 autistic individuals, the age was (M=9.23, SD=7.94), the median was 8, the minimum was 2, and the maximum was 73. There were seven missing values. There were 75 males (72.8%) and 27 females (26.2%), with one missing value [26]. There were 75 Caucasians (72.8%), six Asians (5.8%), three Hispanics (2.9%), 12 Middle Eastern (11.7%), and seven African Americans (6.8%). There were no missing values. In terms of age categories, 18 (17.5%) were in the 1-4-year category, 37 (35.9%) were in the 5-8-year category, 22 (21.4%) were in the 9-12-year category, 15 (14.6%) were in the 13-16-year category, and four (3.9%) were in the 17-73-year category. There were seven (6.8%) missing values. Four subjects were over 17 years old, e.g., 18 years old, 20 years old, 26 years old, and 73 years old [25].

Dataset #4 

Retrospective chart review data were collected from a cohort of 62 autistic individuals who were administered ABA treatment over a five-month snapshot period from August 8, 2023, to January 8, 2024, covering 12 time points (baseline, two weeks, four weeks, six weeks, eight weeks, 10 weeks, 12 weeks, 14 weeks, 16 weeks, 18 weeks, 20 weeks, and 22 weeks) measuring cumulative target behaviors [26]. Behavior technicians assigned to observe specific individuals with autism used real-time data entry techniques, documenting behaviors when they happened. They developed a comprehensive, operational definition for the behavior identified as problematic. They chose continuous measurement systems (frequency and duration tracking) with a portable electronic device, specifically an iPad. The data were then posted online, providing researchers with the necessary information for their analysis and report generation [26].

Sample Demographics

For the sample of 62 autistic individuals, the age was M=8.65 and SD=4.53, the median was eight years, the minimum was two years, and the maximum was 26 years. There were 46 males (74.2%) and 14 females (22.6%), with two (3.2%) missing values [26]. There were 34 Caucasian participants (54.8%), two Asian participants (3.2%), four Hispanic participants (6.5%), 16 Middle Eastern participants (25.8%), and four African American participants. There were two (3.2%) missing values. In terms of age categories, nine (14.5%) were in the 1-4-year category, 21 (33.9%) were in the 5-8-year category, 12 (19.4%) were in the 9-12-year category, seven (11.3%) were in the 13-16-year category, and two (3.2%) were in the 17-26-year category. There were 11 (17.7%) missing values. Two subjects were over 17 years old, e.g., 20 and 26 [26].

Statistical methods 

Statistical Product and Service Solutions (SPSS, version 29.0; IBM Corp., Armonk, NY) was used for all descriptive and reliability statistics [27]. Demographic characteristics were summarized above, including summary statistics for the categorical variables gender, race/ethnicity, continuous variables age (mean and standard deviation, median, range), and individual timepoint variable descriptive statistics. Each valid score in the four datasets was an equally weighted composite score of the number of aggregated general target behaviors mastered, measured at either three, seven, or 12 time points, which were the average of the multiple (three to five behavioral technician) ratings [27]. Internal consistency reliability estimates are presented as Cronbach’s alpha [28,29], inter-item, split-half, and interclass correlation coefficients.

Internal consistency reliability interpretations

The internal consistency reliability of a test is often measured by a correlation coefficient, denoted as α (not to be confused with the α that represents the probability of a Type I error). The value of α can range from 0 to 1, interpreted as follows: If α is greater than or equal to 0.90, the test is considered to have excellent reliability. A test with an α value between 0.70 and 0.90 has good reliability. If α falls within the range of 0.60-0.70, the test has acceptable reliability. The test's reliability is poor if α is between 0.50 and 0.60. A test with an α value less than or equal to 0.50 is considered unacceptable reliability. These ranges serve as a guideline for researchers to evaluate the consistency of their tests.

IRB approval 

Consent was obtained or waived by all participants in this study. This research study retrospectively used data collected from chart reviews for clinical purposes. The study was submitted to the WIRB-Copernicus Group (WCG®IRB) for review and was granted an exemption (#1-1703366-1). The authors declare that this research investigation involves minimal risk and complies with the Belmont Report Regulations 45 CFR 46 2018 Requirements (2018 Common Rule), Section 46, Subpart A Basic HHS Policy for Protection of Human Research Subjects, 46.104 Exempt Research Paragraph d (1), (2), and (2) ii, and 46.117 Documentation of Informed Consent Paragraph c (1) (ii). This study also conformed to the 1964 Declaration of Helsinki guidelines. Note that the Oxford Recovery Center (ORC), which obtained the ClinicalTrials.gov Identifier: NCT06043284, has since rebranded to The Oxford Center (TOC) (additional study ID numbers: OxRS-01-2021).

## Results

Dataset #1

Cronbach's Alpha and Split-Half Reliabilities

Cronbach's alpha for Dataset #1 was 0.916, with seven items indicating excellent reliability. Cronbach's alpha split half Part 1 = 0.777, indicating good reliability; Part 2 = 0.972, indicating excellent reliability; and Guttman split-half coefficient = 0.817, indicating good reliability. The seven items are Targets Mastered Time 1-Baseline, Targets Mastered Time 2-2 Weeks, Targets Mastered Time 3-4 Weeks, Targets Mastered Time 4-6 Weeks, Targets Mastered Time 5-8 Weeks, Targets Mastered Time 6-10 Weeks, and Targets Mastered Time 7-12 Weeks.

Inter-Item Correlation Coefficients

Inter-item correlation coefficients (two-tailed) for the seven timepoint variables ranged from 0.474 to 0.971 and are presented in Table 1.

**Table 1:** Dataset #1 - Inter-Item Correlations

Inter-Item Correlation Variables	Pearson r	p-value	Lower 95% CI Limit	Upper 95% CI Limit
Targets Mastered Time 1-Baseline - Targets Mastered Time 2-2 Weeks	0.711	<0.001	0.592	0.800
Targets Mastered Time 1-Baseline - Targets Mastered Time 3-4 Weeks	0.634	<0.001	0.492	0.743
Targets Mastered Time 1-Baseline - Targets Mastered Time 4-6 Weeks	0.542	<0.001	0.379	0.673
Targets Mastered Time 1-Baseline - Targets Mastered Time 5-8 Weeks	0.525	<0.001	0.356	0.660
Targets Mastered Time 1-Baseline - Targets Mastered Time 6-10 Weeks	0.509	<0.001	0.339	0.647
Targets Mastered Time 1-Baseline - Targets Mastered-Time 7-12 Weeks	0.474	<0.001	0.298	0.619
Targets Mastered Time 2-2 Weeks - Targets Mastered Time 3-4 Weeks	0.785	<0.001	0.691	0.853
Targets Mastered Time 2-2 Weeks - Targets Mastered Time 4-6 Weeks	0.649	<0.001	0.511	0.754
Targets Mastered Time 2-2 Weeks - Targets Mastered Time 5-8 Weeks	0.656	<0.001	0.519	0.760
Targets Mastered Time 2-2 Weeks - Targets Mastered Time 6-10 Weeks	0.639	<0.001	0.499	0.747
Targets Mastered Time 2-2 Weeks - Targets Mastered-Time 7-12 Weeks	0.632	<0.001	0.490	0.741
Targets Mastered Time 3-4 Weeks - Targets Mastered Time 4-6 Weeks	0.866	<0.001	0.803	0.910
Targets Mastered Time 3-4 Weeks - Targets Mastered Time 5-8 Weeks	0.861	<0.001	0.795	0.906
Targets Mastered Time 3-4 Weeks - Targets Mastered Time 6-10 Weeks	0.826	<0.001	0.748	0.882
Targets Mastered Time 3-4 Weeks - Targets Mastered-Time 7-12 Weeks	0.783	<0.001	0.689	0.852
Targets Mastered Time 4-6 Weeks - Targets Mastered Time 5-8 Weeks	0.971	<0.001	0.956	0.981
Targets Mastered Time 4-6 Weeks - Targets Mastered Time 6-10 Weeks	0.930	<0.001	0.896	0.954
Targets Mastered Time 4-6 Weeks - Targets Mastered-Time 7-12 Weeks	0.878	<0.001	0.821	0.918
Targets Mastered Time 5-8 Weeks - Targets Mastered Time 6-10 Weeks	0.970	<0.001	0.954	0.980
Targets Mastered Time 5-8 Weeks - Targets Mastered-Time 7-12 Weeks	0.913	<0.001	0.870	0.942
Targets Mastered Time 6-10 Weeks - Targets Mastered-Time 7-12 Weeks	0.950	<0.001	0.926	0.967

Interclass Correlation Coefficients (ICCs)

ICCs are presented in Table 2. The average measures ICC indicates the reliability of several raters averaged together, used when the average of several ratings is the level of observation in the outcome. It implies excellent reproducibility if you repeat the test several times to calculate the mean value. The average measures value is used when the average of several ratings is the level of observation in the outcome - the average measures interclass correlation equaled 0.916, indicating excellent reliability [30,31].

**Table 2:** Dataset #1 - Intraclass Correlation Coefficients Two-way random effects model where both people effects and measures effects are random.

Intraclass Correlation Type	Coefficient	95% CI Lower Limit	95% CI Upper Limit	F Value	Degrees of Freedom 1	Degrees of Freedom 2	p-value
Single Measures	0.609	0.528	0.692	11.923	89	534	< 0.001
Average Measures	0.916	0.887	0.940	11.923	89	534	< 0.001

The single measures ICC is the reliability coefficient for one single, typical rater. It means fair to good reproducibility if the test is performed on one or several occasions. The single measures rating is used when an individual rating is the level of observation in the outcome. Theoretically, the single measures reliability was 0.609, indicating acceptable reliability if a random single rater was used [30,31].

Dataset #2

Cronbach's Alpha and Split-Half Reliabilities

Cronbach's alpha for Dataset #2 was 0.954, with three items indicating excellent reliability. Cronbach's alpha split half Part 1 = 0.912, indicating excellent reliability; Part 2 = 0.975, indicating excellent reliability; and Guttman split-half coefficient = 0.917, indicating excellent reliability. The items are Targets Mastered Baseline, Targets Mastered 2 Weeks, and Targets Mastered 4 Weeks.

Inter-Item Correlation Coefficients

Inter-item correlations (two-tailed) for three timepoint variables are presented in Table 3. Inter-item correlation coefficients ranged from 0.827 to 0.977.

**Table 3:** Dataset #2 - Inter-Item Correlation Coefficients

Inter-Item Correlation Variables	Pearson r	p-value	Lower 95% CI Limit	Upper 95% CI Limit
Targets Mastered Baseline - Targets Mastered 2 Weeks	0.872	<0.001	0.813	0.914
Targets Mastered Baseline - Targets Mastered 4 Weeks	0.827	<0.001	0.750	0.882
Targets Mastered 2 Weeks - Targets Mastered 4 Weeks	0.977	<0.001	0.966	0.985

ICCs

ICCs are presented in Table 4. The average measures ICC indicates the reliability of several raters averaged together, used when the average of several ratings is the level of observation in the outcome. It implies excellent reproducibility if you repeat the test several times to calculate the mean value. The average measures value is used when the average of several ratings is the level of observation in the outcome - the average measures interclass correlation equaled 0.954, indicating excellent reliability [30,31].

**Table 4:** Dataset #2 - Intraclass Correlation Coefficients Two-way random effects model where both people effects and measures effects are random.

Intraclass Correlation Type	Coefficient	95% CI Lower Limit	95% CI Upper Limit	F Value	Degrees of Freedom 1	Degrees of Freedom 2	p-value
Single Measures	0.875	0.829	0.911	21.923	92	184	<0.001
Average Measures	0.954	0.936	0.968	21.923	92	184	<0.001

The single measures ICC is the reliability coefficient for one single, typical rater. It means fair to good reproducibility if the test is performed on one or several occasions. The single measures rating is used when an individual rating is the level of observation in the outcome. Theoretically, the single measures interclass correlation equaled 0.875, indicating good reliability [30,31].

Dataset #3

Cronbach's Alpha and Split-Half Reliabilities

Cronbach's alpha for Dataset #3 was 0.974, with three items indicating excellent reliability. Cronbach's alpha split-half Part 1 = 0.978, indicating excellent reliability; Part 2 = 0.970, indicating excellent reliability; and Guttman split-half coefficient = 0.935, indicating excellent reliability. The items are Targets Mastered Baseline, Targets Mastered 2 Weeks, and Targets Mastered 4 Weeks.

Inter-Item Correlation Coefficients

Inter-item correlation coefficients (two-tailed) for three timepoint variables are presented in Table 5. Inter-item correlation coefficients range from 0.931 to 0.972.

**Table 5:** Dataset #3 - Inter-Item Correlation Coefficients

Inter-Item Correlation Variables	Pearson r	p-value	95% CI Lower Limit	95% CI Upper Limit
Targets Mastered Baseline - Targets Mastered 2 Weeks	0.972	<0.001	0.958	0.981
Targets Mastered Baseline - Targets Mastered 4 Weeks	0.931	<0.001	0.898	0.954
Targets Mastered 2 Weeks - Targets Mastered 4 Weeks	0.965	<0.001	0.948	0.977

Interclass Correlation Coefficients

Interclass correlation coefficients are presented in Table 6. The average measures ICC indicates the reliability of several raters averaged together, used when the average of several ratings is the level of observation in the outcome. It implies excellent reproducibility if you repeat the test several times to calculate the mean value. The average measures value is used when the average of several ratings is the level of observation in the outcome - the average measures interclass correlation equaled 0.974, indicating excellent reliability [30,31].

**Table 6:** Dataset #3 - Intraclass Correlation Coefficients Two-way random effects model where both people effects and measures effects are random.

Intraclass Correlation Type	Coefficient	95% CI Lower Limit	95% CI Upper Limit	F Value	Degrees of Freedom 1	Degrees of Freedom 2	p-value
Single Measures	0.926	0.897	0.947	38.292	94	188	<0.001
Average Measures	0.974	0.963	0.982	38.292	94	188	<0.001

The single measures ICC is the reliability coefficient for one single, typical rater. It means fair to good reproducibility if the test is performed on one or several occasions. The single measures rating is used when an individual rating is the level of observation in the outcome. Theoretically, the single measures reliability was 0.926, indicating excellent reliability if a random single rater was used [30,31].

Dataset #4

Cronbach's Alpha and Split-Half Reliabilities

Cronbach's alpha for Dataset #4 was 0.980, with 12 items indicating excellent reliability. Cronbach's alpha split half Part 1 = 0.973, indicating excellent reliability; Part 2 = 0.996, indicating excellent reliability; and Guttman split-half coefficient = 0.838, indicating good reliability. The items are Targets Mastered Time 1-Baseline, Targets Mastered Time 2-2 Weeks, Targets Mastered Time 3-4 Weeks, Targets Mastered Time 4-6 Weeks, Targets Mastered Time 5-8 Weeks, Targets Mastered Time 6-10 Weeks, Targets Mastered Time 7-12 Weeks, Targets Mastered Time 8-14 Weeks, Targets Mastered Time 9-16 Weeks, Targets Mastered Time 10-18 Weeks, Targets Mastered Time 11-20 Weeks, and Targets Mastered Time 12-22 Weeks.

Inter-Item Correlation Coefficients

Inter-item correlation coefficients for three timepoint variables are presented in Table 7. Inter-item correlation coefficients ranged from 0.694 to 0.999.

**Table 7:** Dataset #4 - Inter-Item Correlations

Inter-Item Correlation Variables	Pearson r	p-value	95% CI Lower Limit	95% CI Upper Limit
Targets Mastered Time 1-Baseline - Targets Mastered Time 2-2 Weeks	0.960	<0.001	0.935	0.976
Targets Mastered Time 1-Baseline - Targets Mastered Time 3-4 Weeks	0.953	<0.001	0.923	0.972
Targets Mastered Time 1-Baseline - Targets Mastered Time 4-6 Weeks	0.910	<0.001	0.854	0.945
Targets Mastered Time 1-Baseline - Targets Mastered Time 5-8 Weeks	0.841	<0.001	0.748	0.901
Targets Mastered Time 1-Baseline - Targets Mastered Time 6-10 Weeks	0.783	<0.001	0.662	0.864
Targets Mastered Time 1-Baseline - Targets Mastered Time 7-12 Weeks	0.733	<0.001	0.592	0.831
Targets Mastered Time 1-Baseline - Targets Mastered Time 8-14 Weeks	0.695	<0.001	0.539	0.805
Targets Mastered Time 1-Baseline - Targets Mastered Time 9-16 Weeks	0.695	<0.001	0.539	0.805
Targets Mastered Time 1-Baseline - Targets Mastered Time 10-18 Weeks	0.710	<0.001	0.560	0.815
Targets Mastered Time 1-Baseline - Targets Mastered Time 11-20 Weeks	0.717	<0.001	0.569	0.820
Targets Mastered Time 1-Baseline - Targets Mastered Time 12-22 Weeks	0.714	<0.001	0.564	0.819
Targets Mastered Time 2-2 Weeks - Targets Mastered Time 3-4 Weeks	0.987	<0.001	0.978	0.992
Targets Mastered Time 2-2 Weeks - Targets Mastered Time 4-6 Weeks	0.944	<0.001	0.909	0.966
Targets Mastered Time 2-2 Weeks - Targets Mastered Time 5-8 Weeks	0.870	<0.001	0.792	0.92
Targets Mastered Time 2-2 Weeks - Targets Mastered Time 6-10 Weeks	0.806	<0.001	0.697	0.879
Targets Mastered Time 2-2 Weeks - Targets Mastered Time 7-12 Weeks	0.744	< 0.001	0.607	0.838
Targets Mastered Time 2-2 Weeks - Targets Mastered Time 8-14 Weeks	0.701	< 0.001	0.547	0.809
Targets Mastered Time 2-2 Weeks - Targets Mastered Time 9-16 Weeks	0.694	< 0.001	0.538	0.804
Targets Mastered Time 2-2 Weeks - Targets Mastered Time 10-18 Weeks	0.700	< 0.001	0.545	0.808
Targets Mastered Time 2-2 Weeks - Targets Mastered Time 11-20 Weeks	0.702	< 0.001	0.548	0.809
Targets Mastered Time 2-2 Weeks - Targets Mastered Time 12-22 Weeks	0.698	< 0.001	0.542	0.808
Targets Mastered Time 3-4 Weeks - Targets Mastered Time 4-6 Weeks	0.958	< 0.001	0.931	0.975
Targets Mastered Time 3-4 Weeks - Targets Mastered Time 5-8 Weeks	0.885	< 0.001	0.815	0.929
Targets Mastered Time 3-4 Weeks - Targets Mastered Time 6-10 Weeks	0.828	< 0.001	0.729	0.893
Targets Mastered Time 3-4 Weeks - Targets Mastered Time 7-12 Weeks	0.778	< 0.001	0.656	0.861
Targets Mastered Time 3-4 Weeks - Targets Mastered Time 8-14 Weeks	0.741	< 0.001	0.603	0.836
Targets Mastered Time 3-4 Weeks - Targets Mastered Time 9-16 Weeks	0.733	< 0.001	0.592	0.831
Targets Mastered Time 3-4 Weeks - Targets Mastered Time 10-18 Weeks	0.740	< 0.001	0.602	0.835
Targets Mastered Time 3-4 Weeks - Targets Mastered Time 11-20 Weeks	0.741	< 0.001	0.603	0.836
Targets Mastered Time 3-4 Weeks - Targets Mastered Time 12-22 Weeks	0.739	< 0.001	0.598	0.835
Targets Mastered Time 4-6 Weeks - Targets Mastered Time 5-8 Weeks	0.933	< 0.001	0.891	0.959
Targets Mastered Time 4-6 Weeks - Targets Mastered Time 6-10 Weeks	0.891	< 0.001	0.825	0.933
Targets Mastered Time 4-6 Weeks - Targets Mastered Time 7-12 Weeks	0.852	< 0.001	0.765	0.908
Targets Mastered Time 4-6 Weeks - Targets Mastered Time 8-14 Weeks	0.818	< 0.001	0.714	0.887
Targets Mastered Time 4-6 Weeks - Targets Mastered Time 9-16 Weeks	0.804	< 0.001	0.694	0.878
Targets Mastered Time 4-6 Weeks - Targets Mastered Time 10-18 Weeks	0.801	< 0.001	0.690	0.876
Targets Mastered Time 4-6 Weeks - Targets Mastered Time 11-20 Weeks	0.802	< 0.001	0.691	0.877
Targets Mastered Time 4-6 Weeks - Targets Mastered Time 12-22 Weeks	0.800	< 0.001	0.686	0.875
Targets Mastered Time 5-8 Weeks - Targets Mastered Time 6-10 Weeks	0.955	< 0.001	0.926	0.973
Targets Mastered Time 5-8 Weeks - Targets Mastered Time 7-12 Weeks	0.905	< 0.001	0.846	0.942
Targets Mastered Time 5-8 Weeks - Targets Mastered Time 8-14 Weeks	0.869	< 0.001	0.791	0.919
Targets Mastered Time 5-8 Weeks - Targets Mastered Time 9-16 Weeks	0.850	< 0.001	0.762	0.907
Targets Mastered Time 5-8 Weeks - Targets Mastered Time 10-18 Weeks	0.842	< 0.001	0.750	0.902
Targets Mastered Time 5-8 Weeks - Targets Mastered Time 11-20 Weeks	0.840	< 0.001	0.746	0.900
Targets Mastered Time 5-8 Weeks - Targets Mastered Time 12-22 Weeks	0.837	< 0.001	0.742	0.899
Targets Mastered Time 6-10 Weeks - Targets Mastered Time 7-12 Weeks	0.957	< 0.001	0.930	0.974
Targets Mastered Time 6-10 Weeks - Targets Mastered Time 8-14 Weeks	0.919	< 0.001	0.869	0.951
Targets Mastered Time 6-10 Weeks - Targets Mastered Time 9-16 Weeks	0.893	< 0.001	0.828	0.934
Targets Mastered Time 6-10 Weeks - Targets Mastered Time 10-18 Weeks	0.879	< 0.001	0.806	0.925
Targets Mastered Time 6-10 Weeks - Targets Mastered Time 11-20 Weeks	0.874	< 0.001	0.798	0.922
Targets Mastered Time 6-10 Weeks - Targets Mastered Time 12-22 Weeks	0.872	< 0.001	0.794	0.921
Targets Mastered Time 7-12 Weeks - Targets Mastered Time 8-14 Weeks	0.988	< 0.001	0.979	0.993
Targets Mastered Time 7-12 Weeks - Targets Mastered Time 9-16 Weeks	0.972	< 0.001	0.954	0.983
Targets Mastered Time 7-12 Weeks - Targets Mastered Time 10-18 Weeks	0.959	< 0.001	0.933	0.975
Targets Mastered Time 7-12 Weeks - Targets Mastered Time 11-20 Weeks	0.953	< 0.001	0.923	0.971
Targets Mastered Time 7-12 Weeks - Targets Mastered Time 12-22 Weeks	0.952	< 0.001	0.921	0.971
Targets Mastered Time 8-14 Weeks - Targets Mastered Time 9-16 Weeks	0.991	< 0.001	0.985	0.995
Targets Mastered Time 8-14 Weeks - Targets Mastered Time 10-18 Weeks	0.980	< 0.001	0.966	0.988
Targets Mastered Time 8-14 Weeks - Targets Mastered Time 11-20 Weeks	0.973	< 0.001	0.956	0.984
Targets Mastered Time 8-14 Weeks - Targets Mastered Time 12-22 Weeks	0.973	< 0.001	0.955	0.984
Targets Mastered Time 9-16 Weeks - Targets Mastered Time 10-18 Weeks	0.994	< 0.001	0.990	0.996
Targets Mastered Time 9-16 Weeks - Targets Mastered Time 11-20 Weeks	0.990	< 0.001	0.983	0.994
Targets Mastered Time 9-16 Weeks - Targets Mastered Time 12-22 Weeks	0.990	< 0.001	0.983	0.994
Targets Mastered Time 10-18 Weeks - Targets Mastered Time 11-20 Weeks	0.999	< 0.001	0.998	0.999
Targets Mastered Time 10-18 Weeks - Targets Mastered Time 12-22 Weeks	0.999	< 0.001	0.998	0.999
Targets Mastered Time 11-20 Weeks - Targets Mastered Time 12-22 Weeks	0.999	< 0.001	0.998	0.999

Interclass Correlation Coefficients

Interclass correlation coefficients are presented in Table 8. The average measures ICC indicates the reliability of several raters averaged together, used when the average of several ratings is the level of observation in the outcome. It implies excellent reproducibility if you repeat the test several times to calculate the mean value. The average measures value is used when the average of several ratings is the level of observation in the outcome - the average measures interclass correlation equaled 0.980, indicating excellent reliability [30,31].

The single measures ICC is the reliability coefficient for one single, typical rater. It means fair to good reproducibility if the test is performed on one or several occasions. The single measures rating is used when an individual rating is the level of observation in the outcome. Theoretically, the single measures reliability was 0.804, indicating good reliability if a random single rater was used [30,31].

## Discussion

This study aimed to address a gap in the existing literature by examining the psychometric properties of the Catalyst Datafinch data collection application using four distinct research datasets [23-26]. The objective was to evaluate the internal consistency reliability, as determined by Cronbach’s alpha, split-half reliability (both Cronbach’s and Guttman), and inter-item and interclass correlation coefficients. The Catalyst application has been extensively utilized as a digital alternative to traditional paper and pencil methods for tracking skill acquisition and behaviors in individuals with ASD. The results indicated that Cronbach’s alpha, Cronbach’s alpha split-half, Guttman split-half, and inter-item and interclass correlations were predominantly excellent (α ≥ 0.90), with some being good (0.70 ≤ α ≤ 0.90). We observed evidence of alignment when comparing our findings with those of previously published psychometric studies. This consistency provides credence to the robustness of the methodologies employed in these studies, further validating the use of such tools in this area of research. It is noteworthy that the findings of our research align with those of comparable studies.

The median internal consistency reliability for all five MSEL scales ranged from 0.75 to 0.83. The internal consistency for the early learning composite, namely, the four cognitive scales (visual reception, fine motor, receptive language, and expressive language), was between 0.83 and 0.95. Test-retest reliability, with a mean retest time of 11 days (about one and a half weeks), ranged from 0.82 to 0.85 for children 1-24 months (about two years) of age and is less than 0.80 for children 25-56 months (about four and a half years) of age [3].

Zander et al. [32] and Janvier et al. [33] commented on the reliability properties of the ADOS. The median interrater reliability for items across the four modules was 0.74-0.83, with the single ADOS items ranging from 0.23 to 0.94. The total score interrater reliability was 0.85-0.92. Test-retest reliability for the calibrated severity scores of the 608-item ADOS was strong.

Usry et al. reported evidence of excellent inter-rater reliability (ICC=0.95, p<0.001) across the Assessment of Basic Language and Learning Skills (ABLLS-R) scores obtained from a second panel of expert raters [34].

Schmidt et al. [35] and Hobden et al. [36] reported high internal consistency on the ABC, with Cronbach’s alpha ranging from 0.86 to 0.94. The original test-retest reliabilities ranged from 0.96 to 0.99. The whole scale had low interrater reliability with a mean correlation of 0.63. Subsequent studies have shown a range from 0.50 to 0.67 (teacher form) and 0.80 to 0.95 (parent form) [37].

The ADI-R had good internal consistency [38], with test-retest reliability very high at the 0.93-0.97 range. Interrater reliability was as high as the initial study, with multi-rater Kappas ranging from 0.62-0.96 for individual items [39].

The VABS had high internal consistency reliability with split-half reliability for the adaptive behavior composite ranging from 0.93 to 0.97 [40,41], while subdomains were within the 0.80s to 0.90s. Test-retest reliabilities ran mostly from 0.80s to 0.90s [41]. Inter-rater reliabilities ranged from the low 0.70s to the high 0.80s. At the same time, another study found inter-rater coefficients ranging from 0.62 to 0.78 [40,41].

Rimland et al. [42] reported that the total ATEC score had Cronbach alphas of 0.91 and 0.96. The internal consistency of the four ATEC sub-scales was also very high (0.86-0.94 at FU1 and 0.87-0.94 at FU2). Pearson spit half (internal consistency) coefficient for the total ATEC score was 0.942. For the subscales, the coefficients were speech (0.920), sociability (0.836), sensory/cognitive awareness (0.875), and health/physical/behavior (0.815).

The CARS had good internal consistency with Cronbach’s alpha of 0.94 [43,44]. A meta-analysis of research using the CARS between 1980 and 2021 indicated an internal consistency of 0.89 [45]. After a 12-month interval, test-retest reliability for 91 cases was 0.88 for the total score [45]. The inter-rater reliability was found to be 0.71. That same meta-analysis using CARS between 1980 and 2021 found an inter-rater reliability of 0.79.

The Psychoeducational Profile (C-PEP) has internal consistency reliability of the subtests and composites above 0.90 [45,46]. The Cronbach's alphas ranged between 0.92 and 0.98 [47]. The two-week test-retest reliability was 0.94 [48]. C-PEP demonstrated good to excellent inter-rater reliability [47]. The Reynell Developmental Language Scales (RDLS) had no internal consistency reliability estimates publicly available [48]. 

This current research is not without its limitations. The use of four non-random samples limits the scope of the study and restricts the generalizability of the results. The findings may not apply to a broader population or different contexts. The assumption of construct validity in the Catalyst Datafinch application implies that all items are designed to measure the same construct. However, this assumption may not always hold and could potentially impact the accuracy of the results.

Furthermore, the variability in task stimuli, the number of trials, the type of individuals participating, the administration conditions, and the focal task variable across different studies can introduce additional complexity. These factors can influence the outcomes and make it challenging to compare results across studies.

Therefore, while this study provides valuable insights, it is crucial to interpret the findings with these limitations in mind. Future research could address these limitations using a more diverse and randomized sample, ensuring consistent administration conditions and verifying the tools' validity. This would help enhance the robustness and generalizability of the results.

## Conclusions

These findings suggest that the Catalyst Datafinch Applied Behavior Analysis Data Collection Application demonstrates high internal consistency reliability when used with individuals on the autism spectrum. This indicates that the application is a reliable tool for collecting and analyzing behavioral data in this population. The ratings ranged from good to excellent, indicating a high consistency in the measurements obtained through this application. However, it is important to note that these findings, while promising, are part of an ongoing research process. Further studies are necessary to validate these results and ensure the tool’s effectiveness and reliability in diverse settings and populations. Continued research will also help refine the application’s features and functionality, ensuring it remains a valuable resource for those working in the field of ABA. This ongoing commitment to research and validation is crucial in ensuring that the Catalyst Datafinch Application continues to meet the needs of practitioners and individuals with autism.
